# Supplementary material for: CDX2 dose-dependently influences the gene regulatory network underlying human extraembryonic mesoderm development
Source: Biol Open. 2024 Mar 19;13(3):bio060323. doi: 10.1242/bio.060323 (PMC10979512; doi:10.1242/bio.060323)
Supplement: Supplementary information [file biolopen-13-060323-s1.pdf]

Biology Open • Supplementary information

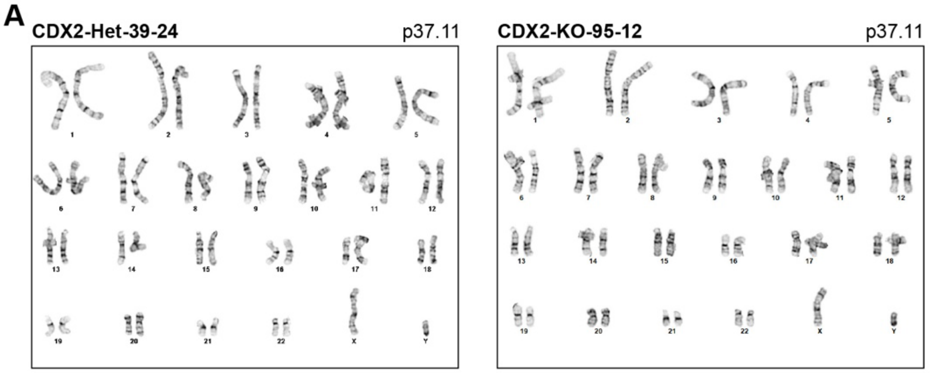

Fig. S1. Karyotyping results from CDX2-Het and CDX2-KO isogenic lines. For WT, see Bulger et al. 2023.

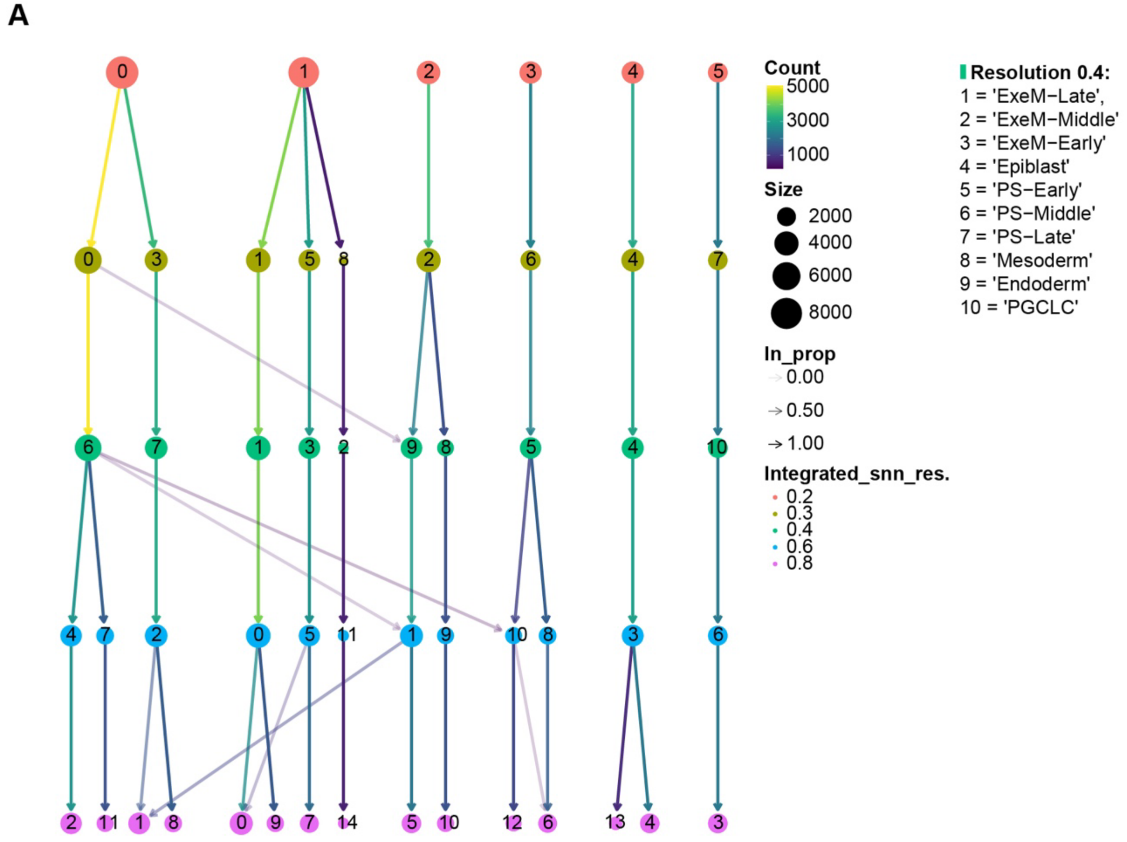

Fig. S2. ClusTree analysis of clusters at 0.2, 0.3, 0.4, 0.6, and 0.8 resolution. A resolution of 0.4 was used for subsequent analyses.

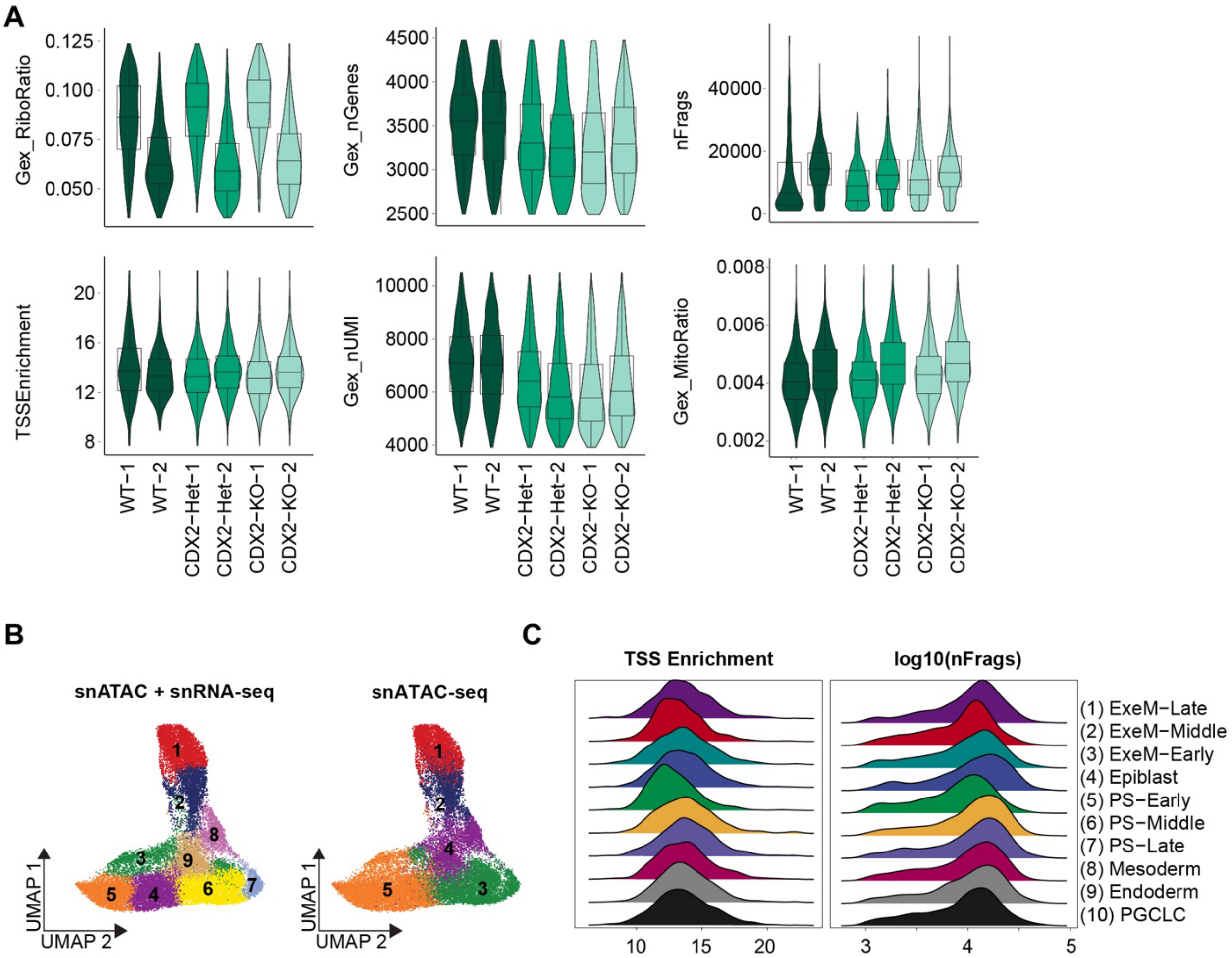

Fig. S3. Quality control parameters for CDX2 snRNA-seq and snATAC-seq data. (**A**) Quality control parameters after filtration, separated by sample. (**B**) TSS enrichment and log10(nFrags), separated by cluster.

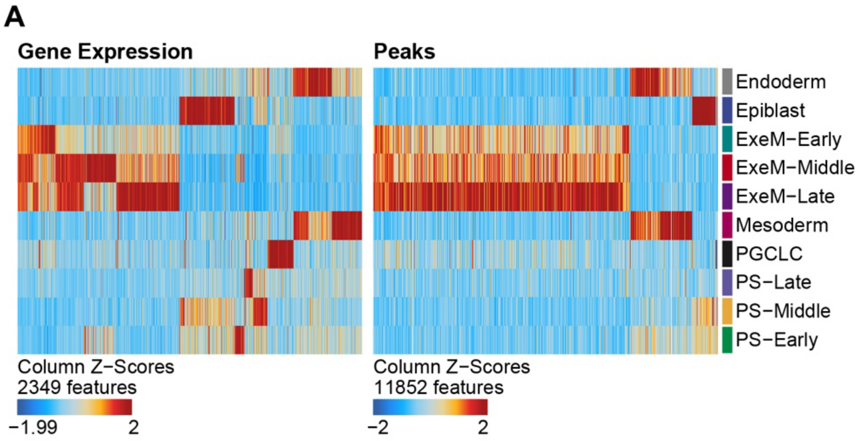

Fig. S4. Gene expression and peak accessibility separated by cluster. The order of rows is based on hierarchical clustering.

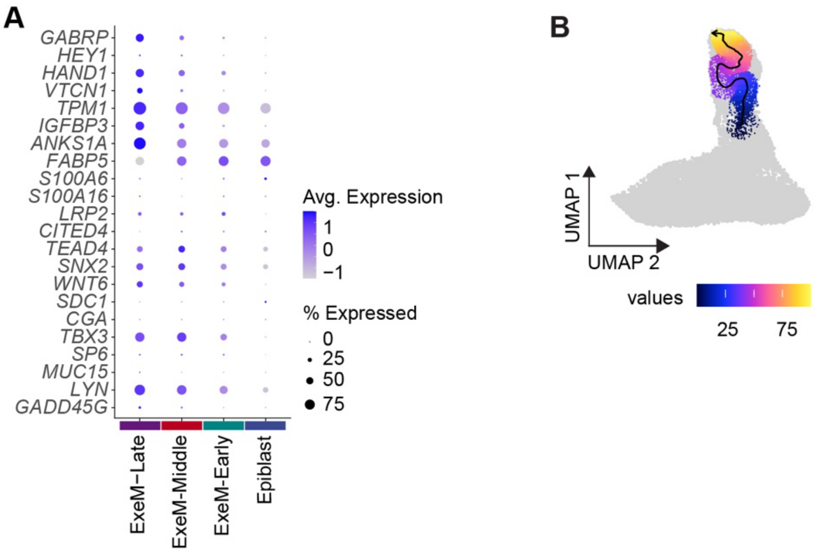

Fig. S5. Extraembryonic mesoderm and trophectoderm marker expression across clusters. (**A**) Dotplot reflecting key markers of amnion and trophectoderm for the extraembryonic and epiblast clusters (C1-C4). (**B**) Trajectory analysis of ExeM-Early, ExeM-Middle, and ExeM-Late (C3-C1).

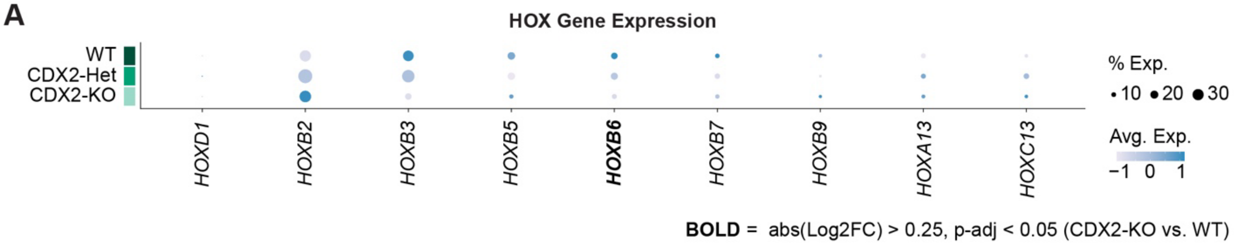

Fig. S6. CDX2 dose-dependently influences downstream HOX expression. (**A**) Gene expression of detectable HOX genes in the ExeM-Late cluster across WT, CDX2-Het, and CDX2-KO. BOLD text indicates Log2FC > 0.25 and p-adj < 0.05 (CDX2-KO vs. WT)

Biology Open • Supplementary information

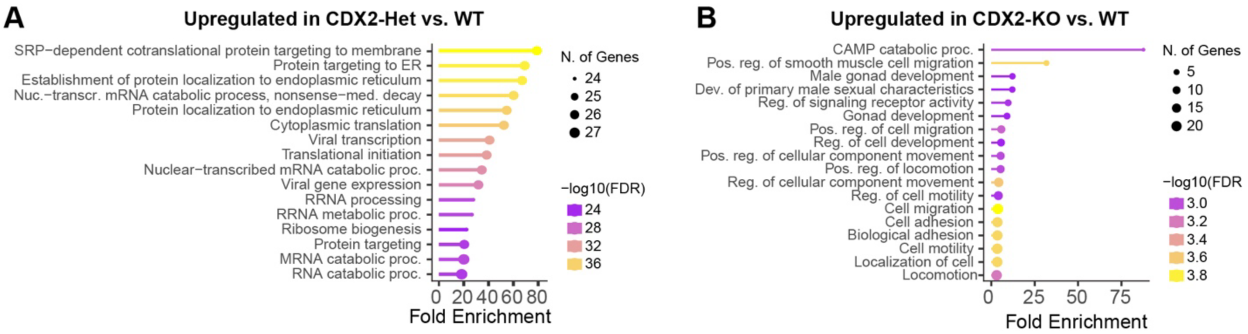

Fig. S7. ShinyGO analysis of CDX2 dosage series. (**A**) ShinyGO analysis of genes upregulated in CDX2-Het vs. WT or (**B**) genes upregulated in CDX2-KO vs. WT (right) (Log2FC > -0.25, p-adj < 0.05).

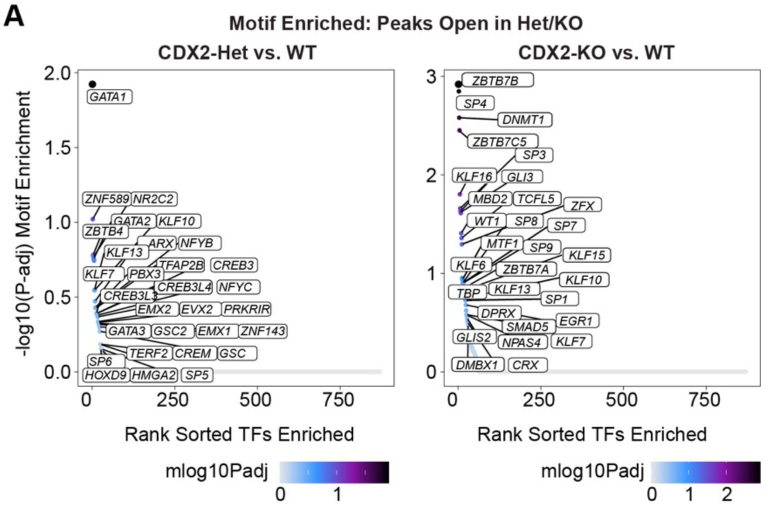

Fig. S8. Motif enrichment in ExE-Late across CDX2 dosage series. (**A**) Motifs enriched in DARs more accessible in CDX2-Het relative to WT or (**B**) CDX2-KO relative to WT.

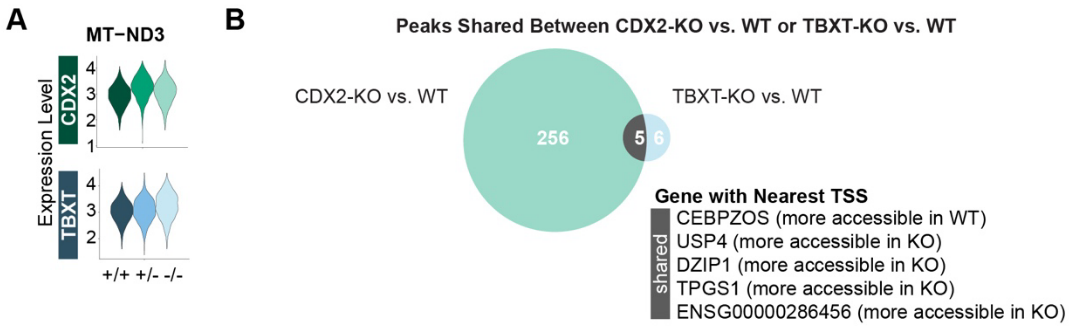

Fig. S9. CDX2-KO and TBXT-KO DEG and Peak comparisons. (A) Gene Expression within the extraembryonic mesoderm cluster for MT-ND3 across CDX2 dosage series (top) or TBXT dosage series (bottom). (**B**) Venn diagram of DARs shared between CDX2-KO vs. WT and TBXT-KO vs. WT (abs(Log2FC) > 0.5, FDR <0.1)

Biology Open • Supplementary information

Table S1. Indel frequency of clonal (#34, 39-24, 95-12) or subclonal (#39-24-2 through 39-24-12) cell populations exposed to the CDX2 sgRNA.

Available for download at
https://journals.biologists.com/bio/article-lookup/doi/10.1242/bio.060323#supplementary-data

Table S2. Overview of differentially expressed genes (DEGs), gene scores, and differential peaks across genotypes within the extraembryonic mesoderm-late cluster.

Available for download at
https://journals.biologists.com/bio/article-lookup/doi/10.1242/bio.060323#supplementary-data

Table S3. ShinyGO analysis.

Available for download at
https://journals.biologists.com/bio/article-lookup/doi/10.1242/bio.060323#supplementary-data

Table S4. Shared differentially expressed genes (DEGs) or Peaks in CDX2 and TBXT dosage series relative to WT.

Available for download at
https://journals.biologists.com/bio/article-lookup/doi/10.1242/bio.060323#supplementary-data
